# Myosin 1F Regulates M1-Polarization by Stimulating Intercellular Adhesion in Macrophages

**DOI:** 10.3389/fimmu.2018.03118

**Published:** 2019-01-10

**Authors:** Zayda L. Piedra-Quintero, Carolina Serrano, Nicolás Villegas-Sepúlveda, José L. Maravillas-Montero, Sandra Romero-Ramírez, Mineko Shibayama, Oscar Medina-Contreras, Porfirio Nava, Leopoldo Santos-Argumedo

**Affiliations:** ^1^Department of Molecular Biomedicine, Cinvestav Zacatenco, Mexico City, Mexico; ^2^Department of Physiology, Biophysics and Neurosciences, Cinvestav Zacatenco, Mexico City, Mexico; ^3^Research Support Network, Universidad Nacional Autónoma de México and Instituto Nacional de Ciencias Médicas y Nutrición “Salvador Zubirán”, Mexico City, Mexico; ^4^Department of Infectomics and Molecular Pathogenesis, Cinvestav Zacatenco, Mexico City, Mexico; ^5^Immunology and Proteomics Laboratory, Mexico Children's Hospital Federico Gómez, Mexico City, Mexico

**Keywords:** myosin 1F, intercellular adhesion, M1-polarization, inflammation, Akt/mTOR/STAT signaling

## Abstract

Intestinal macrophages are highly mobile cells with extraordinary plasticity and actively contribute to cytokine-mediated epithelial cell damage. The mechanisms triggering macrophage polarization into a proinflammatory phenotype are unknown. Here, we report that during inflammation macrophages enhance its intercellular adhesion properties in order to acquire a M1-phenotype. Using *in vitro* and *in vivo* models we demonstrate that intercellular adhesion is mediated by integrin-αVβ3 and relies in the presence of the unconventional class I myosin 1F (Myo1F). Intercellular adhesion mediated by αVβ3 stimulates M1-like phenotype in macrophages through hyperactivation of STAT1 and STAT3 downstream of ILK/Akt/mTOR signaling. Inhibition of integrin-αVβ3, Akt/mTOR, or lack of Myo1F attenuated the commitment of macrophages into a pro-inflammatory phenotype. In a model of colitis, Myo1F deficiency strongly reduces the secretion of proinflammatory cytokines, decreases epithelial damage, ameliorates disease activity, and enhances tissue repair. Together our findings uncover an unknown role for Myo1F as part of the machinery that regulates intercellular adhesion and polarization in macrophages.

## Introduction

Mucosal macrophages play an important role in tissue remodeling, homeostasis, and repair (1, 2). Pathogen-derived molecules or cytokines induce macrophage polarization including M1 and M2 phenotypes (3). In a controlled environment, M1 or (IFN-γ)-type polarization displays bactericidal capacity (4). In contrast, M2 or interleukin-4 (IL-4)-mediated is essential for tissue repair and in the resolution of inflammation (3, 5). M1-macrophage commitment induced by IFN-γ, requires the expression of a specific pool of genes associated with the activation of STAT1 (6–8). In contrast, STAT3 activation downstream of IL-10r/JAK1 has been linked to the so called M2 phenotype and the repression of TNF-α, IL-1β, IL-12, and IFN-γ (9). In addition to STAT signaling, phosphoinositide 3-kinase (PI3K) stimulation is crucial for macrophage polarization (10). For example, recent evidence suggests that PI3K may have an important role in the differentiation of M2-macrophages (3, 11). However, at the inflammation site multiple simultaneous signals are released, consequently, the acquisition of the specialized phenotype displayed by macrophages requires a dynamic response to a specific combination of environmental factors (12, 13). Thus, identifying the machinery responsible for the integration of those inputs is highly relevant for understanding macrophage polarization and physiology.

Myosin family members are proteins that hydrolyze ATP to generate force or directional movement along actin filaments and are implicated in the rearrangement of the cytoskeleton in several cell types (14). Class I myosins also exhibit a C-terminal tail domain that allows them to directly interact with cellular membranes and/or proteins (15). Thus, class I myosins link the actin cytoskeleton with the cell membranes or proteins that are in close proximity (16). Additionally, class I myosins are highly expressed in immune cells; however, their precise functions remain unknown (17). Such specific characteristics make class I myosins good candidates to perform distinctive functions in the immune responses far beyond the actin remodeling. Unconventional myosin 1F (Myo1F), a long tailed class I myosin, is highly expressed in natural killer (NK) cells, macrophages, dendritic cells, and neutrophils (17, 18). However, the role of Myo1F in the immune response mediated by myeloid derived cells has not been completely characterized.

Integrins are single span receptors mediating the cell-extracellular matrix (ECM) adhesion. Ligand-integrin association triggers signal transduction pathways that are implicated in cell cycle regulation, organization of the cytoskeleton, and cell commitment (19, 20). Integrin mobilization and stabilization at the cellular membrane requires controlled mechanical forces mediated by myosin proteins. αVβ3 integrin or vitronectin receptor (CD51/CD61) is highly expressed in differentiated macrophages (21) and plays a key role in the initiation and/or progression of several human diseases (22–25). Integrin-αVβ3 engagement stimulates PI3K/Akt signaling and such process accelerates the commitment of macrophages into a pro-inflammatory phenotype (21, 26). However, the full mechanism by which integrin-αVβ3 regulates macrophage differentiation is not fully understood.

In intestinal inflammation, recruited monocytes commit into a proinflammatory phenotype upon arrival at the inflamed tissue (27). Nevertheless, little is known about the environmental factors and the molecular mechanisms involved in the differentiation of monocytes into colitogenic macrophages (4). Here we report that unconventional Myo1F plays an important role in such process. We demonstrated that Myo1F was induced in colonic macrophages and its presence positively influenced the accumulation of αVβ3-integrin. Such process enhances intercellular adhesion in macrophages and stimulates a proinflammatory phenotype by inducing the activation of ILK/Akt/mTOR signaling which in turn induce STAT1 and STAT3 activation. In macrophages lacking Myo1F the intercellular association mediated by integrin-β3 was reduced; as a consequence, those macrophages failed to fully commit into a M1-phenotype. Interestingly, we also observed that Myo1F upregulation leads to an enhanced secretion and production of IL-1β in macrophages, *in vivo* and *in vitro*. Inhibition of Akt/mTOR or ablation of Myo1F lessened STAT1 and STAT3 activation and reduced the processing and secretion of IL-1β. Consequently, in a model of colitis DSS-induced Myo1F deficiency ameliorated IEC damage and reduced disease symptoms while enhanced epithelial restitution. Thus, our results suggest that ablation of Myo1F prevented the commitment of macrophages into the proinflammatory phenotype and inhibiting Myo1F functions may be a highly effective strategy to ameliorate IBD symptoms.

## Methods

### Mice

Six to seven weeks old C57BL/6J or Myo1F deficient male mice were kept in standard day/night cycles conditions with free access to food and water. All experiments were approved by the Internal Committee for Care and Use of Laboratory Animals guidelines (CICUAL).

### Cell Lines and Bone Marrow-Derived Macrophages (BMM)

J774 macrophages were cultivated in DMEM F-12+ glutaMAX (Gibco) medium supplemented with 10% SFB (biowest) and 1% penicillin/streptomycin (Gibco). Bone marrow-derived macrophages generation was performed as previously described (28). BMM were cultured in DMEM F-12 medium supplemented with 10% SFB, 1% penicillin/streptomycin and GM-CSF (20 ng/ml). On day 7, BMM were stimulated 24 h with LPS (1 μg/ml) + IFN-γ (20 ng/ml) to induce M1-polarization or with IL-4 (20 ng/ml) to induce a M2-phenotype.

### Lentiviral Vector and Cell Transduction

Myo1F was amplified from cDNA obtained from thioglycollate-elicited peritoneal macrophages and cloned in the vector pEGFP-C1(Addgene) using the following primers F-5′-gtcgacatgggcagagcgctttcact-3′, and R-5′-ggatcctcagatccatagtttcctgg-3′. Next, Myo1F-GFP was subcloned in the pLEX vector (Addgene) using the restriction sites: SnaBI and BamHI. Lentiviral particles for transduction were produced using Trans-Lentiviral Packaging System #TLP5912 (Dharmacon) accordingly to the manufacturer's instructions. Positive cells were selected with Puromicyn (10 μg/ml).

### Aggregation Assay

BMM and transduced J774 or Raw264.7 were collected. Next, cells were resuspended in serum free DMEM F-12 media. Cell suspension was placed in 1.5 ml microfuge tubes precoated with 2% BSA and rotated on a gyratory shaker at 37°C for 45 min. Integrin-β3 inhibitor [1 μM] (Cilegitide trifluouroacetic acid salt, Sigma-Aldrich SML1594) or vitronectin [1.5 μg/ml] (Advanced BioMatrix cat#5051) was added. Aggregation was stopped by adding 2% (vol/vol) glutaraldehyde. The extent of aggregation was assessed by microscope analysis.

### Isolation of Colonic Cells and Flow Cytometry

Colonic cells were isolated and staining for flow cytometry as described previously (29). Immune cells were identified as follows: macrophages (MΦs), CD45^+^IAb^+^CD11b^+^F4/80^+^; dendritic cells (DCs), CD45^+^IAb^+^CD11c^+^CD103^+^ and monocytes (Mos), CD45^+^CD11b^+^Ly6C^+^Gr1^−^. Flow cytometry assay was performed on CytoFLEX cytometer (Beckman-Coulter) and analyzed with the CytExpert software.

### Antibodies and Reagents

Primary antibodies were as follows: Myo1F HPA055242 (Sigma-Aldrich), GAPDH/sc-322 (Santa Cruz Biotechnology®), TNF-α #11948, pSTAT1-Tyr701 #9167, pS6 #15967, pAkt-Ser473 #4060, pAkt-Ser308 #4056, Akt1 #2967 from Cell Signaling Technology®, CD86-PE-CY5 #15-0862-82 (eBioscience), and CD80-PE #553769 (BD pharmingen). Alexa conjugated antibodies and affiniPure goat and rabbit anti-horseradish peroxidase (HRP) were obtained from Thermo Fisher Scientific and Jackson Immunoresearch, respectively. 4′,6-Diamidino-2-phenylindole (DAPI) sc-3598 from Santa Cruz Biotechnology. Recombinant mouse IFN-γ from PeproTech was dissolved in 0.002% mouse serum albumin (MSA; Sigma-Aldrich) and used at 2.5 μg/kg of mice weight. Lipopolysaccharide from *Escherichia coli* 0111:B4 (Sigma-Aldrich) was dissolved in 0.002% mouse serum albumin and used at 100 μg/mice. AZD8055 [(5-{2,4-Bis[(3S)-3-methylmorpholin-4-yl]pyrido[2,3-d]pyrimidin-7-yl}-2-methoxyphenyl)methanol] from Astra Zeneca was dissolved in DMSO and use at 20 nM. Dextran sulfate sodium (DSS) YD318041799 from Carbosynth was dissolved at 2.5% in tap water.

### RT-PCR Assays

Real time-PCR for iNOS expression in BMM from WT and Myo1F^−/−^ was performed as previously reported (30).

### *In vivo* IFN-γ/LPS Injection

Mice were randomly divided into groups. IFN-γ/LPS was administered intraperitoneally and control mice received carrier alone (Mouse Serum Albumin, 0.02% in PBS). Five hours post-injection mice were euthanized, and colons processed for immunofluorescence and Western blot assays.

### Colitis Induction in Mice

Six to eight weeks old WT or Myo1F KO mice were housed in standard conditions (temperature, light, 5 animals per cage, etc) with free access to food and water. On day 0 colitis was induced by administration of 2.5% (wt/vol) of DSS (molecular mass 40 kDa, Carbosynth, CA) dissolved in tap water. Control group were separated and received tap water alone. Mice were monitored daily for changes in weight, presence of blood in stool and appearance of diarrhea. On the day of sacrifice, colons were assessed for weight, length, and processed for histology (31). Colon was processed as previously reported by us for Immunofluorescence and Western Blot analysis (32). Restitution and wound healing assays in a colitis model were performed as previously reported (33).

### Immunofluorescence, Histology, and Western Blot Assays

Immunofluorescence, histology and Western blot experiments of colonic mucosa were performed as previously described (32). For WB, samples were collected in Ripa lysis buffer sonicated and cleared by centrifugation. Protein concentration was determined using a BCA protein assay and equal amount of proteins separated in polyacrylamide gels. Immunofluorescence studies were performed in cells grown on transparent filter inserts or in 20 μm tissue sections. Fixation was carried out with PFA 4% Wt/Vol and permeabilization performed with 100% methanol at −20°C for 20 min. Samples were incubated with primary antibodies overnight at 4°C. Images were taken on an LSM 510 confocal microscope (Zeiss). H&E staining was performed as previously describe (34).

### Cytokine Release Analysis

IL-1β and IL-6 release was quantified by ELISA (Biolegend #432605 and #431305) or LEGENDplex assay (BioLegend #740150) on supernatants obtained from cell cultures or from colonic explants.

### Statistical Analysis

Statistical analysis was performed using Prism 6.0 (GraphPad Software San Diego, CA). Data were analyzed by one-way ANOVA and two-tailed Student's *t*-test, a *p*<*0.05* was considered statistically significant.

## Results

### Enhanced Intercellular Adhesion Properties in M1 Macrophages Are Regulated by Myo1F

**Figure 1:**
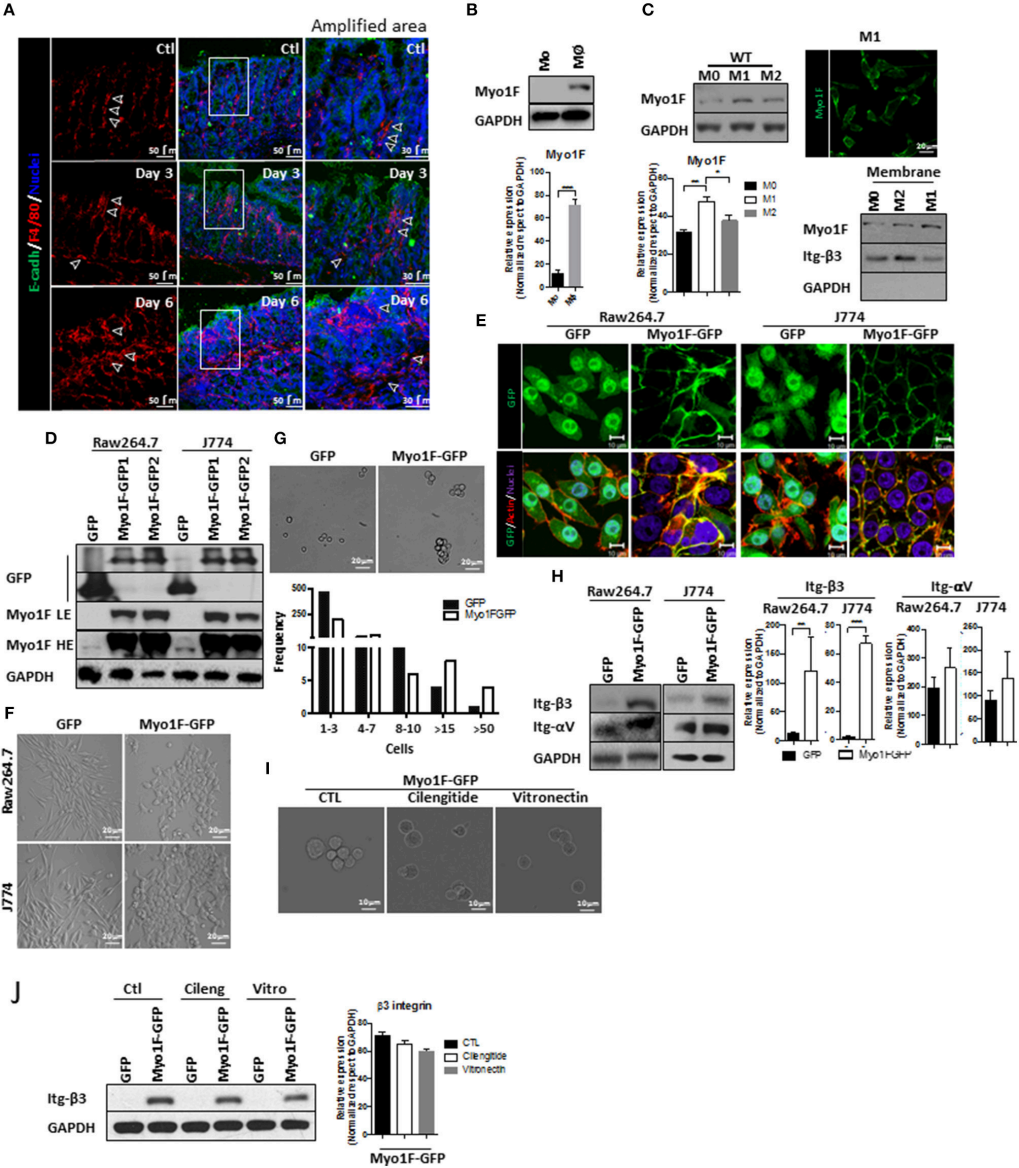
Myo1F enhances intercellular adhesion integrin-α*νβ*3-mediated in macrophages. **(A)** Representative immunofluorescence staining of E-cadherin (green) and F4/80 (red) in the colonic mucosa of C57BL/6J mice that were induced to colitis. Macrophages in colonic epithelium were identified as F4/80^+^ and epithelial cells are defined by the expression of E-cadherin. Colitis was induced by administration of 2.5% DSS in drinking water. Control animals received water alone. Nuclei were stained with Dapi (blue). Bar = 50 μm. White arrows defined areas with macrophages. Amplified images of the areas marked by a white square are shown. Bar = 30 μm. *n* = 10. **(B)** Monocytes (Mo) from bone marrow obtained from C57BL/6J mice were differentiated to macrophages (Mφ) as describe in materials and methods. Myo1F was analyzed by western blot. GAPDH was used as loading control. *n* = 3. Densitometric analyses obtained from those results are shown as graphs. ***p = 0.0005. **(C)** Myo1F was analyzed by western blot in cell lysates of BMM obtained from C57BL/6J mice that were differentiated into M0, M1, and M2 phenotype. GAPDH was used as loading control. Densitometric analyses obtained from those results are shown as graphs. *p = 0.05, **p = 0.01. Immunofluorescence staining for Myo1F (Green) was carried out in M1 macrophages obtained from C57BL/6J. *n* = 3. Membrane bound Myo1F was evaluated in BMM differentiated to M0, M1, and M2. Cytosolic and membrane fractions were prepared as previously reported (36). Integrin β3 marks membrane fraction and GAPDH marks cytosolic fraction. **(D)** Western blot for Myo1F and GFP was performed on cell lysates of RAW264.7 and J774 cells overexpressing Myo1F-GFP or GFP. Macrophages were transduced as described in material and methods. GAPDH was used as loading control. LE = Low exposure. HE = High Exposure. *n* = 5. **(E)** Cellular distribution of Myo1F was evaluated by confocal microscopy in transduced macrophages expressing Myo1F-GFP or control GFP. GFP = green. Actin = red. Nuclei = blue. Bar = 10 μm. *n* = 5. **(F)** Bright field images of RAW264.7 and J774 cells overexpressing Myo1F-GFP or GFP plated in flat surface. Bar = 20 μm. *n* = 5. **(G)** Cell adhesion assay was carried out with RAW264.7 overexpressing Myo1F-GFP or GFP. Bright field shows representative images of the assay. Cells were maintained in suspension for 30 min in serum free media in mild agitation. Bar = 20 μm. *n* = 5. Graphical representation of the cell aggregation is shown. **(H)** Western blot for αν and integrin-β3 was performed on cell lysates of RAW264.7 and J774 cells overexpressing Myo1F-GFP or GFP. GAPDH was used as loading control. Densitometric analyses obtained from those results are shown as graphs. **p = 0.01, ***p = 0.0005. **(I)** Cell adhesion assay was carried out with RAW264.7 overexpressing Myo1F-GFP or GFP in presence of cilentigide or vitronectin. Bright field shows representative images of the assay. Bar = 10 μm. *n* = 3. **(J)** Cell adhesion assay was carried out with RAW264.7 overexpressing Myo1F-GFP or GFP in presence of cilentigide or vitronectin. Bright field shows representative images of the assay. Bar = 10 μm. *n* = 3. Densitometric analyses obtained from those results are shown as graphs.

Mucosal macrophages play an important role in the maintenance of intestinal homeostasis (35). Therefore, we investigated the presence of macrophages in the colonic mucosa of control and colitic mice. In control mice, macrophages formed a defined line bordering the colonic crypts. In contrast, during colitis displayed an epithelial-like shape and formed aggregates immediately below the epithelium (Figure 1A). Furthermore, in an adhesion assay where BMM were maintained in suspension for 30 min in serum free media (37), IFNγ/LPS-induced proinflammatory macrophages formed large aggregates. In contrast, anti-inflammatory macrophages obtained after IL-4 stimulation remained as single cells (Figure S1A). These results suggested that pro and anti-inflammatory macrophages displayed different adhesion properties.

Cytoskeleton rearrangements and integrin localization downstream of myosin proteins regulate cellular adhesion (38–40). Therefore, we investigated if Myosin 1F (Myo1F) an unconventional myosin expressed mainly in myeloid lineages (17, 41) was affected during macrophage differentiation. As shown in Figure 1B a clear increase in Myo1F was noticed in monocytes that were differentiated into macrophages. Furthermore, Myo1F protein levels were augmented in BMM induced to M1 phenotype when compared with their M0 and M2 counterparts (Figure 1C). Myo1F in M1-macrophages was enriched at the cellular borders (Figure 1C; Figure S1B). Therefore, a putative link between Myo1F presence and the adhesive properties of M1 macrophages was investigated. To address this question J774 and RAW264.7 cells were stably transduced with GFP or Myo1F-GFP lentivirus (Figures S1C,D; Figure 1D). It is noteworthy to mention that endogenous Myo1F was detected in J774 and RAW264.7 cells (Figure 1D). Immunofluorescence staining demonstrated enrichment of Myo1F-GFP at the cell borders but GFP alone was observed at cytosol and nucleus (Figure 1E). Furthermore, Myo1F-GFP transduced cells were flatter with epithelioid-like phenotype (Figures 1E,F) and (42) and GFP expressing cells displayed a spindled-like phenotype. Also, Myo1F-GFP growing in flat surfaces formed small colonies resembling the observed for epithelial cells, in contrast GFP transduced cells appeared scatter in the whole platting surface (Figures 1E,F).

In addition, in an adhesion assay the solely overexpression of Myo1F resulted in the formation of large aggregates consisting of more of >15 RAW264.7 cells. In contrast, RAW264.7 cells GFP transduced appeared as single cells or forming small aggregates ≤ 8–10 cells (Figure 1G). Similar results were observed in J774 cells (**Data not shown**). Myo1F was playing a direct role in the intercellular adhesion in macrophages, because M1 BMM lacking Myo1F remained as single cells in an adhesion assay (Figure S1A). We speculated that upregulation of integrins-αV and β3 in Myo1F-GFP expressing cells could be responsible for the enhanced adhesives observed in RAW264.7 cells and J774 cells (Figure 1H). To test such possibility, integrin-αVβ3-function was inhibited with cilengitide or soluble monomeric vitronectin, two broadly used inhibitors (43–45). Both inhibitors strongly reduced the cellular aggregation triggered by Myo1F overexpression without dramatically affecting integrin-β3 protein levels (Figures 1I,J) or αV integrin (**Data not shown**). Thus, our results strongly suggest that Myo1F positively regulates the intercellular adhesion in macrophages by increasing the adhesion mediated by integrin-α*νβ*3.

### Myo1F Stimulates Pro-inflammatory Phenotype in Macrophages

**Figure 2:**
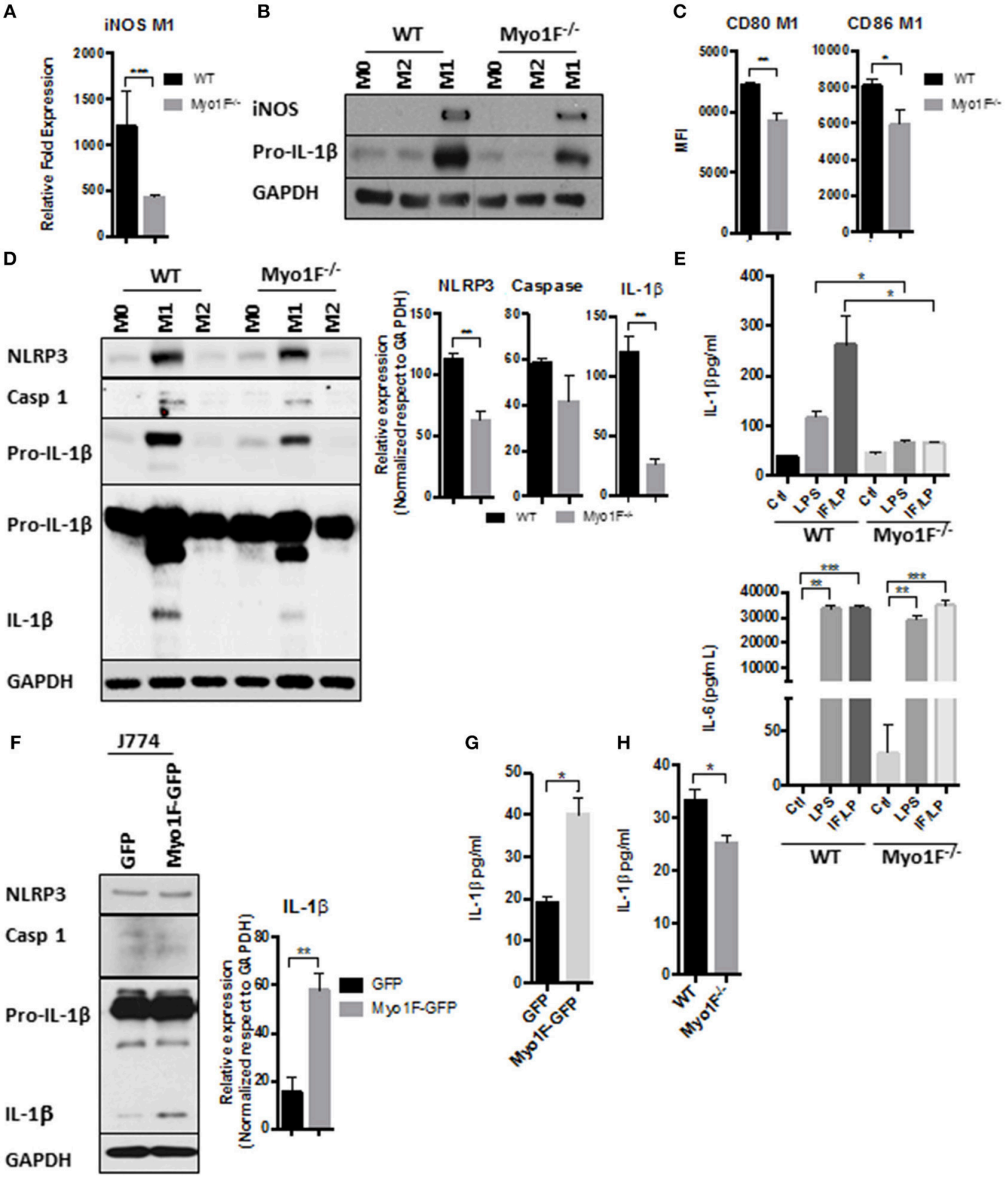
Myo1F is required to stimulate a pro-inflammatory phenotype in macrophages. **(A)** iNOS expression was analyzed by RT-PCR in WT and Myo1F deficient bone marrow-derived macrophages differentiated into M1 phenotype. M1 phenotype was induced by IFN-γ/LPS (20 ng/ml; 1 μg/ml) stimulation. *n* = 3. Results are given as mean values ± SD. ***p = 0.0005. **(B)** iNOS and pro-IL-1β were analyzed by western blotting cell lysates of WT and Myo1F deficient bone marrow-derived macrophages differentiated into M0, M1, or M2 phenotype. M1 phenotype was induced by IFN-γ/LPS (20 ng/ml; 1 μg/ml) stimulation and M2 was obtained by IL-4 (20 ng/ml) exposition. GAPDH was used as loading control. *n* = 3. **(C)** Expression of CD80 and CD86 was analyzed by flow cytometry in WT and Myo1F deficient bone marrow-derived macrophages differentiated into M1 phenotype and expressed as Mean Florescence Intensity. M1 phenotype was induced by IFN-γ/LPS (20 ng/ml; 1 μg/ml) stimulation. *n* = 3. Results are given as mean values ± SD. *p = 0.05, **p = 0.01. **(D)** NLRP3, Caspase 1, proIL-1β, and IL-1β were analyzed by western blotting cell lysates of WT and Myo1F deficient bone marrow-derived macrophages differentiated into M1 or M2 phenotype. M1 phenotype was induced by IFN-γ/LPS (20 ng/ml; 1 μg/ml) stimulation and M2 was obtained by IL-4 (20 ng/ml) exposition. GAPDH was used as loading control. *n* = 3. Densitometric analyses obtained from those results are shown as graphs. **p = 0.01. **(E)** Secretion of IL-1β and IL-6 in supernatants of WT and Myo1F^−/−^ derived BMM was performed by ELISA assay. LPS and IFN-γ/LPS stimulation was carried out for 5 h. Graphs are derived from independent experiments carry out by duplicate. *n* = 3. Results are given as mean values ± SEM. *p = 0.05, **p = 0.01, ***p = 0.0005. **(F)** Western blotting for NLRP3, Caspase 1 and IL-1β in J774 cells overexpressing Myo1F or GFP under homeostatic conditions. GAPDH was used as loading control. *n* = 3. Densitometric analysis obtained from IL-1β is shown as graph. **p = 0.01. **(G)** Secretion of IL-1β in supernatants of J774 cells overexpressing Myo1F or GFP was performed by ELISA assay. Graphs are derived from independent experiments carry out by duplicate. *n* = 3. Results are given as mean values ± SEM. *p = 0.05. **(H)** Quantification of IL-1β release was analyzed in colonic explants of WT and Myo1F deficient mice stimulated with IFN-γ/LPS. Inflammatory stimulus was administered for 5 h. IL-1β was quantified by ELISA. Graphs are derived from three independent experiments. *n* = 6. Results are given as mean values ± SEM. *p = 0.05.

Integrin-β3 engagement stimulates M1 polarization in macrophages (21, 26, 46). Therefore, we analyzed the presence of M1 polarization markers in macrophages lacking Myo1F. To this end the expression of IL-1β, iNOS, TNF-α, CD80, CD86 (30, 47, 48) was studied in BMM of Myo1F^−/−^ and WT origin. As shown in Figures 2A–C and Figure S2A, the presence and expression of iNOS, IL-1β, TNF-α, CD80, and CD86 was decreased in BMM lacking Myo1F, suggesting a reduction in M1-polarization in the absence of Myo1F. The absence of TNFα, IL-1β, and iNOS in M0 and M2 macrophages demonstrated the efficiency and specificity of the treatments. Additionally, high levels of pSTAT6, a well-known repressor that limits the inflammatory response (49), were detected in BMM from Myo1F^−/−^ origin when compared with WT macrophages. In agreement with this last finding, the solely overexpression of Myo1F reduced the presence of the repressor (Figure S2B). Also, iNOS was augmented in J774 transduced with Myo1F-GFP when compared with J774 GFP-expressing cells. However, Arginase-1 an M-2 marker displayed the opposite behavior (Figure S2C).

Next, we investigated in a more detailed manner the processing of IL-1β in BMM lacking Myo1F. Therefore, we first analyzed the presence of RELA (p65), an essential component of NFκB, a transcription factor highly involved in the expression of the components of the inflammasome (50). As shown in Figure S2D very low levels of pp65 (active) and p65 were detected in M1 macrophages lacking Myo1F in comparison with BMM of WT origin. In fact, pp65 and p65 detected in M1 macrophages Myo1F deficient were more similar to the levels expressed by M2-macrophages of WT and Myo1F^−/−^ origin. Furthermore, because the overexpression of Myo1F also increased the levels of pp65 and p65 we believe that Myo1F positively regulates the activation of NFκB in macrophages.

In agreement with these findings, NLRP3, Pro-IL-1β, and IL-1β was strongly reduced in BMM lacking Myo1F when compared with M1 macrophages from WT origin (Figure 2D). However, as expected (51), caspase-1 was not affected in the absence of Myo1F. M0 and M2 macrophages from both, WT or Myo1F^−/−^ origin, displayed low levels of NLRP3, Pro-IL-1β, IL-1β, and caspase-1 when compared with M1 macrophages demonstrating specificity and efficiency of the treatments. Furthermore, IL-1β release was induced in WT BMM exposed to LPS or IFN-γ/LPS and the effect was significantly attenuated in BMM lacking Myo1F. However, in non-stimulated BMM cells from Myo1F^−/−^ and WT origin was similar (Figure 2E). Contrary to IL-1β, the release of IL-6, another proinflammatory cytokine produced by macrophages (52), was boosted at basal state in BMM lacking Myo1F when compared with its WT counterparts, but its secretion was stimulated at similar extent by LPS or IFN-γ/LPS in, both BMM of WT or Myo1F^−/−^ origin (Figure 2E). Thus, these results strongly suggest a direct role of Myo1F in the production and secretion of IL-1β.

To corroborate our findings, we next analyzed NLRP3, Caspase-1, Pro-IL-1β, and IL-1β in cell lysates of J774 GFP and J774 Myo1F-GFP cells. As shown in Figures 2F,G, Myo1F overexpression induced a small but constant accumulation of NLRP3 and augmented the processing and secretion of IL-1β in J774 cells. However, its presence failed to increase pro-IL-1β or caspase-1. Furthermore, in line with our previous results the secretion of IL-1β that resulted from colonic explants harvested from mice IP injected with IFN-γ/LPS was strongly reduced in colonic explants of Myo1F KO mice (Figure 2H). Thus, in conjunction our results suggest that Myo1F is important for the transformation of macrophages into a M1-phenotype and in the processing and secretion of IL-1β by those cells.

### Myo1F Regulates Akt/STAT Signaling in Macrophages

**Figure 3:**
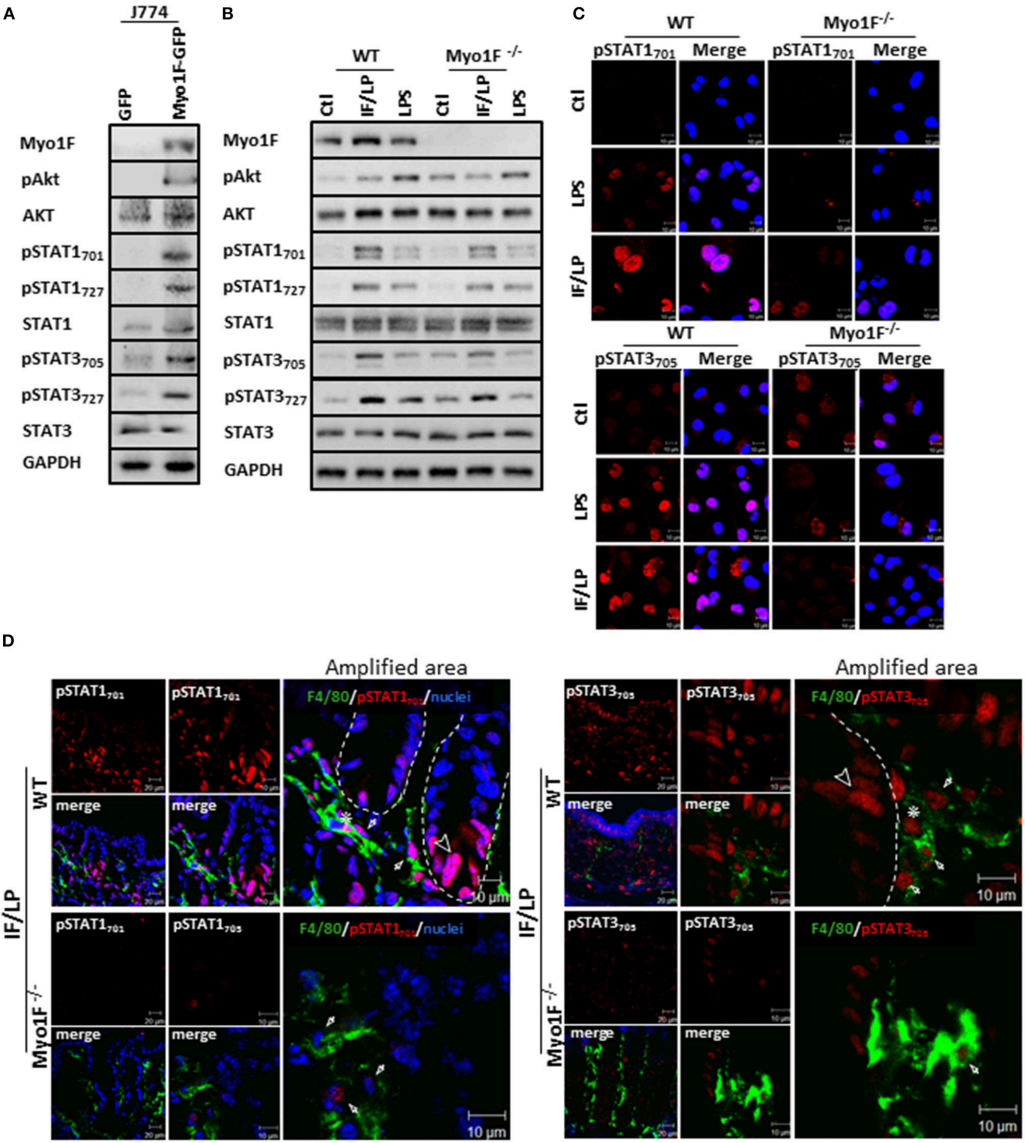
Myo1F mediates activation and cellular localization of Akt/STAT signaling in macrophages. **(A)** Myo1F, pAkt_473_, AKT, pSTAT_701_, pSTAT_727_, STAT1, pSTAT3_705_, pSTAT3_727_, and STAT3 were analyzed in cell lysates of J774 cells expressing Myo1F-GPF or GFP. Cells were platted at confluence for 12 h before lysis. GAPDH was used as loading control. *n* = 3. **(B)** Myo1F, pAkt_473_, Akt, pSTAT_701_, pSTAT_727_, STAT1, pSTAT3_705_, pSTAT3_727_, and STAT3 were studied in WT or Myo1F^−/−^ BMM. Cell lysates were carried out in macrophages control or stimulated for 5 h with IFN-γ (20 ng/ml)/plus LPS (1 μg/ml) stimulation or LPS alone (1 μg/ml). GAPDH was used as loading control. *n* = 3. **(C)** Immunofluorescence staining for pSTAT1 and pSTAT3 (red) was performed in WT or Myo1F^−/−^ BMM platted in glass coverslips. Cell were fixed after 5 h stimulation with IFN-γ (20 ng/ml)/plus LPS (1 μg/ml), LPS (1 μg/ml) or carrier alone (Ctl). Nuclei were stained with Dapi (blue). Scale bar 10 μm. **(D)** Representative image of immunofluorescence staining for F4/80 (green) and pSTAT_701_ (red) or pSTAT3_705_ (red) in colonic epithelium from WT and Myo1F deficient mice intraperitoneally injected with IFN-γ/LPS for 5 h. Nuclei were stained with Dapi (blue). Bar = 20 μm. Amplified areas of the images are shown. Bar = 10 μm. *n* = 5.

Next, we investigated the mechanism by which Myo1F stimulates the commitment of proinflammatory macrophages. Akt signaling has been linked to M1 polarization in macrophages downstream of integrin-αVβ3 (21). Therefore, the status Akt in J774 cells transduced with GFP or Myo1F-GFP was analyzed. As shown in Figure 3A, Myo1F overexpression induced activation of Akt (S473; from now on pAkt) in J774 cells without affecting Akt protein levels. This process was likely a consequence of the upregulation and relocation of ILK to the cellular membrane (Figures S3A,B, **White arrow**), a kinase known to activate Akt downstream of αVβ3 integrin (53). Interestingly, Myo1F overexpression also enhanced the phosphorylation of STAT1 (Y701, S727) and STAT3 (Y705, S727), two transcription factors implicated in the commitment of macrophages into a proinflammatory phenotype (54). The process occurs without affecting STAT1 or STAT3 protein levels (Figure 3A).

To corroborate our findings, we next analyzed the status of Akt and STAT signaling in BMM derived from WT or Myo1F deficient animals that were stimulated with LPS or IFN-γ/LPS. As shown in Figure 3B and Figure S4A increased phosphorylation in the components of both signaling pathways was observed in WT macrophages challenged with both stimuli. However, the phosphorylation of pAkt_473_, pSTAT_701_, pSTAT_727_, pSTAT3_705_, and pSTAT3_727_ that was induced after IFN-γ/LPS stimulation was greatly attenuated in BMM cells lacking Myo1F. In contrast, when BMM were stimulated with LPS alone, only the phosphorylation of pSTAT3_727_ was reduced in the absence of Myo1F (Figure S4A). No changes in total Akt, STAT1 or STAT3 were induced by any stimuli. Next, we evaluated the localization of STAT1 and STAT3 in the conditions previously described. IF analysis demonstrated that LPS or IFN-γ/LPS stimulation induced nuclear accumulation of pSTAT1_701_ and pSTAT3_705_ in WT macrophages. However, nuclear translocation of both molecules was greatly reduced in macrophages lacking Myo1F (Figure 3C). It is noteworthy to mention that in unstimulated BMM lacking Myo1F we noticed high levels of pAkt and pSTAT3 (at nuclei). However, no activation of pSTAT1 was observed (Figures 3B,C). Because of these results we next evaluated the activation of STAT signaling in the colonic mucosa of WT and Myo1F deficient mice administered intraperitoneally with IFN-γ/LPS (55, 56). As expected enhanced accumulation of pSTAT1 and pSTAT3 in nuclei of epithelial (Arrowhead) and macrophages (F4/80-positive cells) **(Asterisk)** present at the colonic mucosa was induced by the stimulus. Nuclear staining of pSTAT1 and pSTAT3, however, was clearly reduced in macrophages lacking Myo1F (Figure 3D, **Arrow**). Thus, our findings strongly suggest that Myo1F is necessary for the proper compartmentalization and activation of Akt and STAT signaling in colonic macrophages during proinflammatory stimuli.

### Myo1F Regulates STAT Signaling in Macrophages via PI3K/Akt/mTORC1

**Figure 4:**
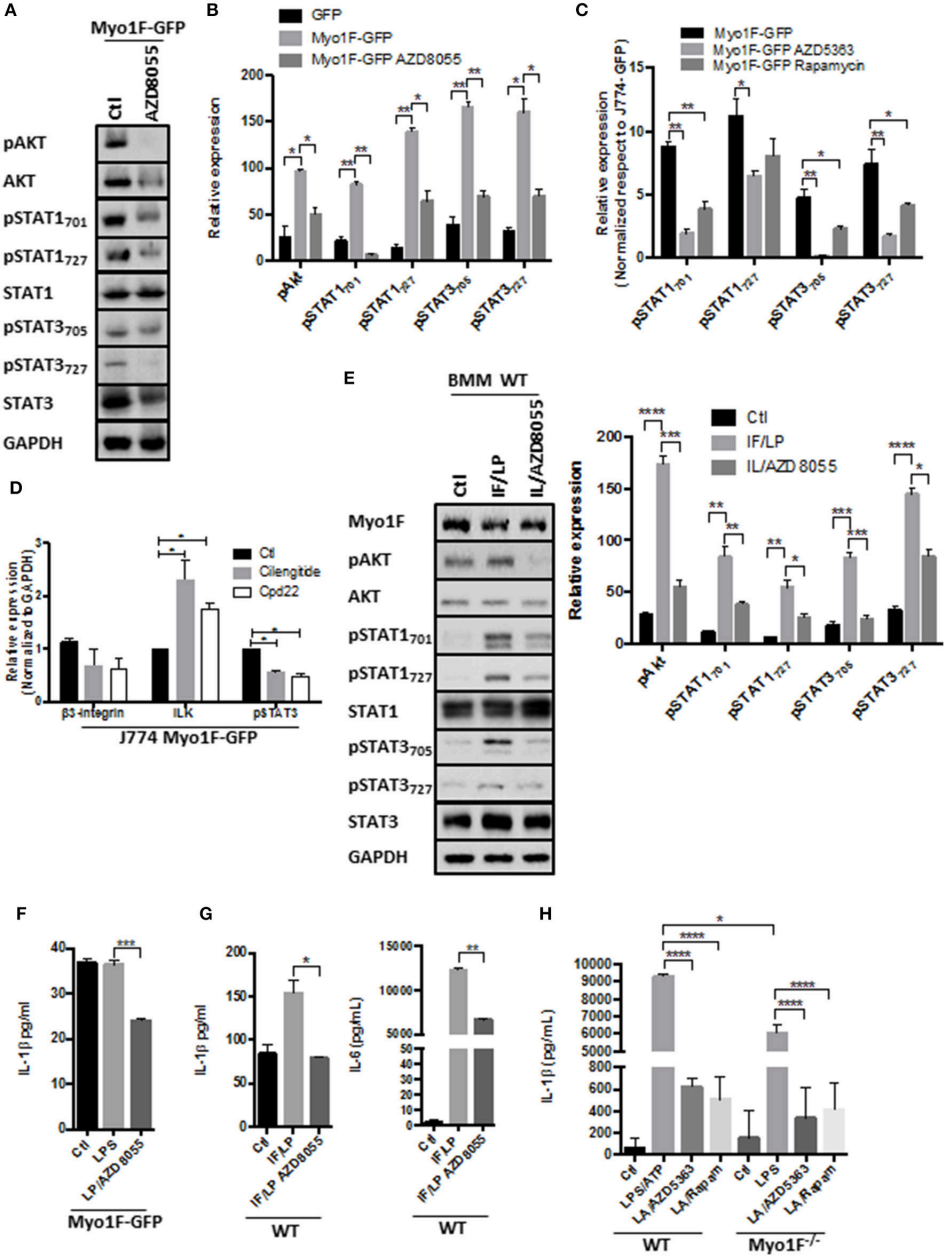
PI3K/Akt/mTORC1 activates STAT signaling in macrophages downstream of the Myo1F. **(A)** pAkt_473_, Akt, pSTAT_701_, pSTAT_727_, STAT1, pSTAT3_705_, pSTAT3_727_, and STAT3 levels were investigated in J774 Myo1F-GFP cells. AZD8055 (20 nM) was administered. GAPDH was used as loading control. **(B)** Densitometric analyses obtained from **(A)** are shown as graphs. *p = 0.05, **p = 0.01. **(C)** Densitometric analysis for pSTAT_701_, pSTAT_727_, STAT1, pSTAT3_705_, pSTAT3_727_, and STAT3 in J774 cell overexpressing Myo1F-GFP. AZD8055 (20 nM) or Rapamycin (20 nM) were administered for 5 h. Values were normalized to J774-GFP activity. Graph = Mean ± SD. *p = 0.05, **p = 0.01. **(D)** Integrin-β3, ILK, and pSTAT3 were analyzed in cell lysates of J774 cells overexpressing Myo1F-GFP. Cilengitide (Integrin-β3 inhibitor; 1 μM) and Cpd22 (ILK inhibitor; 4 μM) were administered for 6 h. Graph corresponding to densitometric analysis derived from three independent experiments is shown. *n* = 3. *p = 0.05. **(E)** Western blot for pAkt, Akt, pSTAT_701_, pSTAT_727_, STAT1, pSTAT3_705_, pSTAT3_727_, and STAT3 was performed in bone marrow-derived macrophages from WT origin. IFN-γ (20 ng/ml)/LPS (1 μg/ml) was carried out (IF/LP) for 5 h. AZD8055 (20 nM) was administered 30 min before IF/LP stimulation. GAPDH was used as loading control. *n* = 3. Densitometric analyses obtained from those results are shown as graphs. *p = 0.05, **p = 0.01, ***p = 0.0005, ****p < 0.0001. **(F)** Quantification of IL-1β release by ELISA was investigated in J774 cells overexpressing Myo1F-GFP stimulated with LPS (1 μg/ml) for 5 h. AZD8055 (20 nM) was administered 30 min before LPS stimulation. Graphs are derived from three independent experiments. *n* = 6. Results are given as mean values ± SEM. ***p = 0.0005. **(G)** Quantification of IL-1β and IL-6 secretion by ELISA assay was performed in bone marrow-derived macrophages from WT origin. BMM cells were stimulated with IFN-γ (20 ng/ml)/LPS (1 μg/ml) for 5 h. AZD8055 (20 nM) was administered 30 min before IFN-γ/LPS stimulation. Graphs are derived from independent experiments carry out by duplicate. *n* = 3. Results are given as mean values ± SEM. *p = 0.05, **p = 0.01. **(H)** IL-1β release was analyzed in supernatants obtained from BMM of WT or Myo1F^−/−^ origin. LPS stimulation (1 μg/ml) was carried out for 4 h follow by 1 h of ATP (freshly prepared, 5 mM) induction. AZD5363 (20 nM) or Rapamycin (20 nM) was administered 30 min before LPS stimulation. Graph = Mean ± SD. *p = 0.05, ****p < 0.0001.

PI3K/Akt/mTORC1 signaling can drive M1-polarization via phosphorylation of STAT1 and STAT3 at the residue serine 727 (57, 58). Therefore, we analyzed the phospho status of Akt, STAT3, and STAT1 in J774 Myo1F-GFP cells that were treated with the mTOR inhibitor, AZD8055. As shown in Figure 4A the administration of AZD8055 reduced the phosphorylation of Akt, STAT1_727_, and STAT3_727_ in J774 Myo1F-GFP cells. As a consequence, STAT1_701_ and STAT3_705_ are decreased. Furthermore, a more detailed analysis of our results demonstrated that by inhibiting mTOR function the phosphorylation levels of Akt_473_, STAT1_727_, STAT3_727_, STAT1_701_, and STAT3_705_ in Myo1F-GFP transduced cells was comparable to the observed in non-stimulated J774-GFP cells (Figure 4B). Furthermore, the administration of AZD8186, a PI3K inhibitor, yielded similar results (Figure S4B). No changes in total STAT1, STAT3 or Akt were detected in any of the treatments. Reduced phosphorylation of Akt demonstrated the efficiency of the treatments. To investigate the role of specific components of the PI3K/Akt/mTORC1 signaling in the activation of STAT proteins we next evaluated the effect of inhibiting Akt (AZD5363) and mTORC1 (Rapamycin) in J774 Myo1F-GFP cells. As shown in Figure 4C, Akt and mTORC1 inhibition prevented serine and tyrosine phosphorylation in STAT1 and STAT3 proteins. In agreement with these findings Myo1F OE in J774 cells induced mTORC1 activation, as shown by the hyperphosphorylation of S6 (59). In the contrary, in M1-macrophages lacking Myo1F the phosphorylation of S6 was attenuated (Figure S4C). However, it is noteworthy to mention that our results clearly demonstrated that Akt inhibition was more efficient than rapamycin to inhibit STAT signaling. Thus, taking together our results strongly suggest that PI3K/Akt/mTORC1 signaling positively regulates STAT activation in macrophages.

In agreement with our hypothesis that Akt/STAT signaling is activated by integrin-α*νβ*3/ILK downstream of Myo1F we observed that cilengitide and cpd22 reduced STAT3 activation in J774 Myo1F-GFP. Both treatments reduced integrin-β3 protein levels, but surprisingly they increased ILK (Figure 4D). Remarkably, this mechanism requires the secretion of unknown factors that could be acting autocrinally, because the administration of BfA reduced the activation of Akt and STAT3 (Figure S4D). To corroborate our findings, we next analyzed the phosphorylation of Akt, STAT1 and STAT3 in BMM derived from WT animals that were stimulated with IFN-γ/LPS in the presence of AZD8055. As shown in Figure 4E, pAkt_473_, pSTAT1_727_, pSTAT3_727_, pSTAT1_701_, and pSTAT3_705_ presence increased after IFN-γ/LPS treatment but was reversed after mTOR inhibition. Decreased pAkt levels demonstrated the efficiency of the treatment. No changes in Akt, STAT1, or STAT3 were observed (Figure 4E).

Because PI3K/Akt/mTORC1 signaling also stimulates acute secretory responses (60), we next investigated the effect of AZD8055 in the secretion of IL-1β induced by Myo1F. As shown in Figure 4F, AZD8055 prevented the release of IL-1β induced by LPS in J774 cells transduced with Myo1F-GFP and also decreases the release of IL-1β in BMM of WT origin that were stimulated with IFN-γ/LPS (Figure 4G). Importantly this effect was not specific for IL-1β because the secretion of IL-6 induced by IFN-γ/LPS was attenuated after mTOR inhibition. Next, we investigated the effect of PI3K/Akt/mTORC1 in the release of IL-1β induced by ATP (61). To this purpose BMM of WT or Myo1F^−/−^ origin were stimulated with LPS and freshly prepared ATP. As shown in Figure 4H by inhibiting Akt or mTORC1 in BMM of WT we prevented the strong release of IL-1β that was induced by LPS/ATP. As expected from our previous results lack of Myo1F significantly reduced IL-1β secretion and this effect was further enhanced in the presence of the inhibitors (Figure 4H). Thus, our results suggest that downstream of Myo1F, the PI3K/Akt/mTORC1 signaling is necessary to stimulate STAT proteins but also instigates the secretion of IL-1β.

### Myo1F Deficiency Reduces Intestinal Epithelial Damage and Enhances Epithelial Restitution in a Model of DSS-induced Colitis

**Figure 5:**
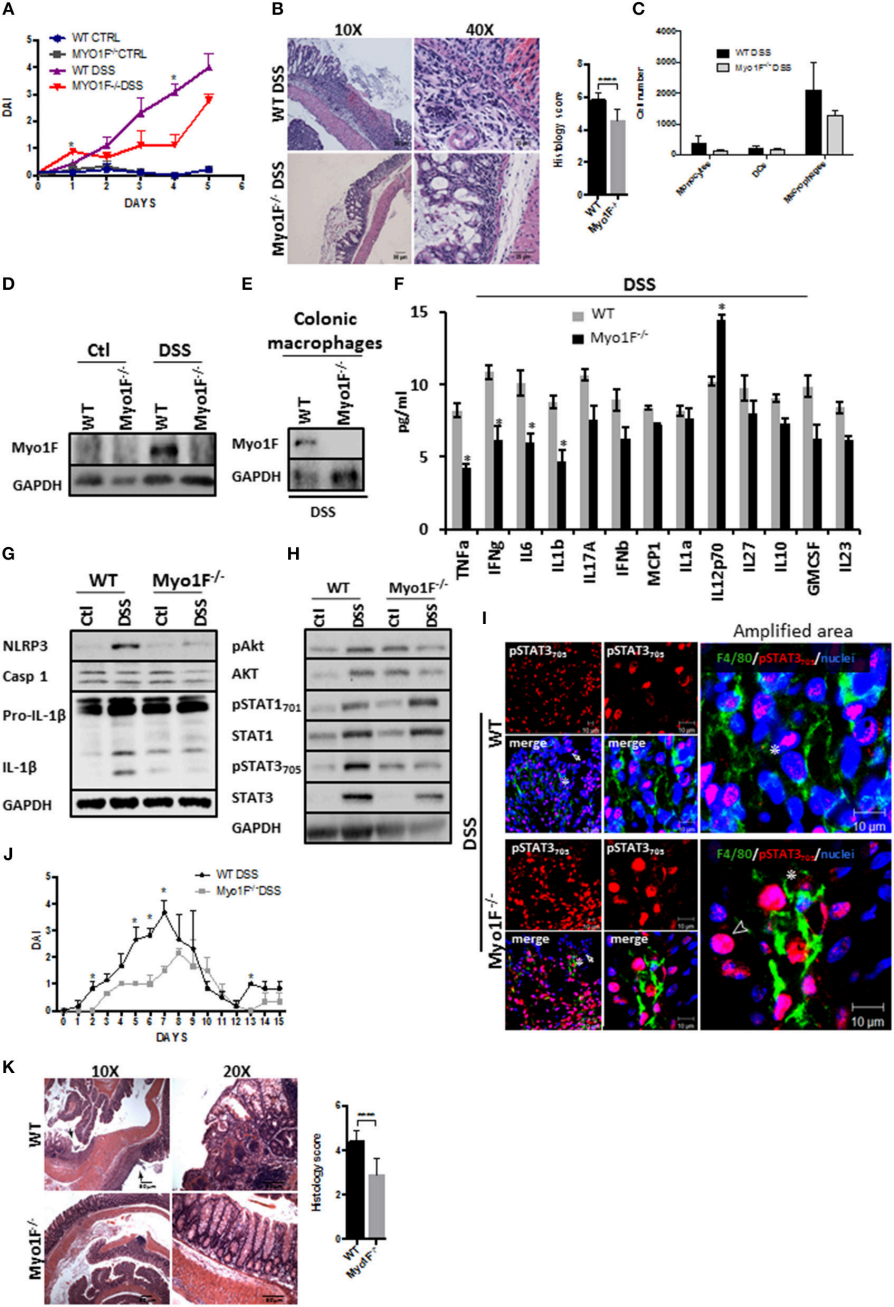
Reduced intestinal epithelial damage and enhanced epithelial restitution in the colonic mucosa of Myo1F^−/−^ was observed after colitis induction. **(A)** Disease Activity Index of WT and Myo1F deficient mice induced to colitis with 2.5% DSS in drinking water. All the results are derived from independent experiments carry out by duplicate. *n* = 5 mice per group. *p = 0.05. **(B)** Hematoxylin & eosin staining of colonic sections obtained from colitic WT and Myo1F deficient mice. DSS 2.5% was administered in drinking water for 5 days. Bar = 50 μm. Amplification area is shown. Bar = 25 μm. Histological score was determined from H&E stain. ****p = 0.0001. **(C)** Quantification of myeloid cells isolated from colon of WT and Myo1F^−/−^ mice induced to colitis with DSS for 5 days. Purification and quantification were performed by Flow cytometry. All the results are derived from independent experiments carry out by duplicate. **(D)** Myo1F presence was evaluated by western blotting in colon cell lysates of WT and Myo1F deficient mice induced to colitis with DSS. Treatment was carried out for 5 days. Control animals received only drinking water. GAPDH was used as loading control. *n* = 5. **(E)** Myo1F protein levels were analyzed in colonic macrophages isolated from colitic mice as described in materials and methods. GAPDH was used as loading control. *n* = 3. **(F)** Cytokine and chemokine secretion was analyzed by using a multiplex assay in colonic explants of WT and Myo1F deficient mice that were induced to colitis for 5 days. Results are given as mean values ± SEM. *p = 0.05. **(G)** NLRP3, Caspase 1 and IL-1β was evaluated in cell lysates of colonic epithelium obtained from WT or Myo1F deficient mice under control condition or after 5 days of treatment with DSS. GAPDH was used as loading control. *n* = 5. **(H)** pAkt_473_, Akt, pSTAT_701_, STAT1, pSTAT3_705_, and STAT3 were analyzed in lysates of colonic tissue from WT and Myo1F deficient mice induced to colitis with DSS. GAPDH was used as loading control. *n* = 3. **(I)** Representative image of immunofluorescence staining for F4/80 (green) and pSTAT3_705_ (red) in colonic epithelium from WT and Myo1F deficient mice induced to colitis. Nuclei were stained with Dapi (blue). Bar = 20 μm. Amplified areas of the images are shown. Bar = 10 μm. *n* = 5. Disease activity index **(J)** and histological changes **(K)** were analyzed in WT and Myo1F^−/−^ that were induced to recuperation after colitis induction. Hematoxylin & eosin staining was carried out as described in materials and methods. Bar = 50 μm. Arrow marks ulcerated areas. All the results are derived from independent experiments carry out by duplicate. *n* = 5 mice per group. *p = 0.05, ****p = 0.0001. Histological score was determined from H&E stain.

We next analyzed the functional relevance of macrophage polarization induced by Myosin1F in a model of inflammation. To this end, we investigated the development of colitis mediated by the administration of DSS in Myo1F deficient mice. At basal conditions, no gross abnormalities or histological changes in the crypt architecture were detected in the colon of Myo1F KO mice when compared with its WT counterpart (Figures S5A,B). Furthermore, the number of monocytes, macrophages and dendritic cells present at the colonic tissue of WT and Myo1F deficient animals were equivalent (Figure S5C). However, DSS treatment induced a more severe colitis in WT than in Myo1F KO mice as shown by weight loss, DAI and histological analysis (greater mucosal ulcerations, increased edema and more infiltration) (Figures 5A,B; Figure S5D). Additionally, the reduction of colon length induced by the treatment was similar in both animals (Figure S5E). No substantial changes in any parameter were detected in WT or Myo1F deficient mice challenged with water (Figure 5A). Next, we investigated if Myo1F deficiency affected the recruitment of some myeloid cells to the colon. The number of monocytes, macrophages and dendritic cells that were present in the inflamed mucosa of WT and Myo1F deficient mice was similar (Figure 5C). Furthermore, as shown in Figures 5D,E, Myo1F was enriched in the colonic mucosa of WT mice that were induced to colitis and more specifically in colonic macrophages. However, colitis induction failed to stimulate the production of Myo1F in the colonic mucosa of Myo1F^−/−^ animals and also was absent in colonic macrophages of Myo1F^−/−^ colitic mice. In contrast to Myo1F, minimal changes in integrin-α*νβ*3 and ILK were displayed in the colonic mucosa of control WT and Myo1F^−/−^ mice and both molecules were increased at similar extent after colitis induction in both animals (Figure S5F). Thus, in agreement with our previous results these findings strongly suggested that Myo1F deficiency ameliorates the symptoms of DSS-mediated colitis by a mechanism that does not impairs recruitment of monocytes, macrophages and dendritic cells into the inflamed area.

Proinflammatory cytokines secreted by M1-macrophages including TNF-α, IL-1, IL-6, IL-12, Type I IFN, CXCL1-3, CXCL-5, and CXCL8-10 (5) have been associated to mucosal damage during DSS-induced colitis and also are known to prevent epithelial restitution (62, 63). Therefore, we screened cytokine secretion in colonic explants of WT and Myo1F deficient animals that were induced to colitis for 5 days. We found that TNF-α, IFN-γ, IL-6 and IL-1β were significantly decreased in Myo1F^−/−^ mice as compared to WT animals, but IL12p70 was upregulated. No changes in IL-17A, IFN-α, MCP1, IL-1α, IL-27, IL-10, GM-SCF, or IL2-3 were noticed (Figure 5F). Furthermore, in agreement with our *in vitro* findings NLRP3, pro-IL-β, and IL-1β were reduced in the colonic mucosa of colitic Myo1F deficient when compared with DSS-treated WT animals (Figure 5G). No changes in caspase-1 were detected. Next, we analyzed Akt, STAT1 and STAT3 in colonic cell lysates of WT and Myo1F^−/−^ animals that were induced to colitis. In agreement with our previous results, phosphorylation of Akt and STAT3 was reduced in the mucosa of colitic Myo1F deficient mice when compared with WT animals (Figure 5H). However, STAT1 phosphorylation was similar. No changes in total Akt, STAT1, or STAT3 were observed in any condition. In addition, immunofluorescence analysis demonstrated total absence of pSTAT1 staining at nuclei of mucosal cells in, WT or Myo1F^−/−^ colitic mice (Figure S5G). However, pSTAT3 staining was observed in IECs (**Arrow**) and in macrophages (**Asterisk**) present at the lamina propria. A more detailed analysis (**amplified area**) demonstrated a clear reduction in nuclear pSTAT3 in colonic macrophages (**Asterisk**) of Myo1F^−/−^ mice (Figure 5I). It is noteworthy to mention that in the colonic mucosa of Myo1F deficient mice nuclear pSTAT3 accumulates in non-epithelial cells that were negative for F4/80 in control and inflammatory conditions (Figure 5I, Arrowhead). Those cells were absent or non-detected in the colonic mucosa of WT animals. Thus, our findings suggest that Myo1F enhances activation of Akt and STAT3 signaling in colonic macrophages during colitis.

Therefore, we next analyzed long-term effects of Myo1F depletion in a mouse model of chronic DSS-induced colitis. Upon the administration of DSS, minor effects were noticed in Myo1F KO mice in contrast; WT animals rapidly lost weight and increased disease symptoms (Figure 5J and Figure S5H). Furthermore, upon sacrifice ulcerated lesions spread throughout the colon of WT animals but not in the colon of Myo1F deficient mice (Figure 5K). These results demonstrate that Myo1F reduces repair following DSS-mediated injury. Thus, taken together all our results support the hypothesis that Myo1F induces M1-polarization in macrophages during colitis by enhancing its intercellular adhesion via integrin-α*νβ*3 which in turns triggers PI3K/Akt/mTORC1/STAT signaling.

## Discussion

Disruption of intestinal homeostasis by proinflammatory cytokines contributes to development and establishment of chronic pathologies, such as inflammatory bowel diseases (IBD) (64, 65). In this study, we showed that Myo1F expressed in inflammatory macrophages in the colonic mucosa of colitic mice stimulates the production and secretion of IL-1β via stimulation of Akt and STAT signaling. In the absence of Myo1F the production of IL-1β by macrophages decreased in the mucosa of colitic mice and this process resulted in accelerated epithelial injury reparation. Our findings also show that Myo1F is involved in the macrophage polarization to a phenotype that resembles the colitogenic lineage described in response to DSS challenge (4).

Myo1F overexpression could stimulate such phenomena by triggering activation of STAT1 and STAT3 signaling by stimulating the axis α*νβ*3/ILK/PI3K/Akt/mTORC1, a process well-documented in another models (21, 46, 66, 67). However, our findings highlighted several unknown facts about macrophage polarization, for example during colitis DSS–induced pSTAT1 was never detected at the nuclei of macrophages or IECs, but in contrast, nuclear staining of pSTAT3 was promoted in both cell types in the mucosa of WT animals. Thus, we speculated that in macrophages, during M1-polarization, the transcriptional activity of STAT1 could be transiently stimulated by Myo1F or proinflammatory stimuli. In that context, epigenetical modifications induced during STAT1 activation will allow STAT3 to elicit a proinflamatory phenotype rather than the antiinflamatory functions associated with the molecule (68, 69). In fact, it has been shown that DNA-binding of STAT1 and IRF-1 sometimes primes chromatin for recruitment of other transcription factors to specific promoters and enhancers, such as the ones located in TNF, IL-6, and IL-12B (70). A key process of this last mechanism implies sustained activation of STAT1 (71) a process that has been achieved in our model by overexpressing Myo1F. Therefore, we could speculate that fewer molecules of STAT1 are recruited to specific promoters in the nucleus and therefore are undetectable at nuclei using immunofluorescence. Another plausible option dictates that a complete rearrangement of the chromatin was induced by the transitory occupancy of the promoters by the molecule. However, it cannot be ruled out the possibility that transient activation of STAT1 stimulates the expression of proteins (e.g., NF-κB) that are necessary for M1-polarization. In that context, sustained activation of STAT3 will privilege the formation of STAT3/NF-κB complexes rather than STAT3/STAT3 homodimers. Such process will stimulate transcriptional production of both, IL-6 and IL-1β (72, 73), a process that will lead to the results observed here. Furthermore, the alternate functions of pSTAT1 aside of its role as a transcription factor open the possibility that sustained activation of STAT1 is necessary to regulate a different biological process, such as mitophagy (74).

Despite the previous controversy our results clearly showed that Myo1F presence enhances STAT1 and STAT3 phosphorylation a process that improves the interaction of STAT proteins with cognate docking sites (75). The mechanism by which Myo1F enhances and sustains the activation of STAT proteins in macrophages needs to be investigated but it could be mediated by the direct interaction of Myo1F with Syk and ITAM-containing adaptor proteins (71). The protein-protein interaction domains identified in Myo1F could allow the protein to create such interactions (76). However, the results presented here clearly demonstrated that PI3K/Akt/mTORC1 pathway also plays an important role in this process. The mechanism reported for such process involves direct phosphorylation of the serine 727 by the mammalian target of rapamycin complex 1 (mTORC1) in both molecules, STAT1 and STAT3 (59, 77). However, it could be more complex because the inhibition of several components in the PI3K/Akt/mTORC1 pathway resulted in the reduction in the phosphorylation of STAT1 and STAT3 at tyrosine 701 and 705, respectively.

The activation of PI3K signaling by Myo1F could have far more implications in the biogenesis of proinflammatory macrophages than only stimulating the activation of STAT1 and STAT3. In fact, it could be a key component in the regulation of movement, secretion and protein synthesis in macrophages. In that context, we also observed that absence of Myo1F reduces the secretion and processing of IL-1β probably by controlling exocytosis, inflammasome assembly, NF-κB function, synthesis and degradation of the proteins (78–81). The exact mechanism involved in the biosynthesis of active IL-1β by Myo1F needs to be investigated, but our results strongly suggest that Akt and mTORC1 play an important role in the process. Interestingly, based in our results we can suggest that Myo1F is necessary for modulating the secretion of IL-1β via unconventional mechanisms (82, 83) that could rely in the activation of IL-1β via autophagy and/or the storage of IL-1β in intracellular vesicles (84). However, because the release of IL-1β induced by ATP stimulation (61, 85) was attenuated in the absence of Myo1F or in the presence of Akt or mTORC1 inhibitors, we cannot ruled out the possibility that autophagy (84) or protein synthesis are necessary for cytokine biogenesis in macrophages. Therefore, activation of PI3K/Akt/mTORC1 signaling could be more important than previously thought in the production and release of proinflammatory cytokines.

Activation of STAT3 by mTORC1 promotes survival and cancer stem-like cell properties (58, 59) a phenotype not observed in macrophages expressing Myo1F. However, Myo1F-deficient macrophages displayed an M2-like phenotype characterized by active Akt, STAT3, and STAT6 (49, 86). Therefore, we hypothesized that activation of STAT1 downstream of Myo1F could be an essential factor in the polarization of M1 macrophages. Thus, the mechanism employed by Myo1F to activate STAT1 via mTORC1 could represent the switch triggering such process. Furthermore, because Myo1F participates in macrophage differentiation, the genetic footprint of WT and Myo1F KO macrophages could help to elucidate the mechanisms and the machinery implicated in the polarization of macrophages.

The mechanism by which Myo1F enhances IEC damage during colitis could be directly related to increase in the production/secretion of proinflammatory cytokines by macrophages. In this context, we and other have shown that IFN-γ, TNF-α, IL-6, and IL-1β stimulate several signaling pathways that stimulate cell death and reduce cell proliferation of IECs at the mucosa of colitic mice. The amelioration of symptoms and the fast recovery in the epithelial damage observed in the mucosa of Myo1F^−/−^ mice that were induced to colitis support those facts (87). Thus, targeting Myo1F in macrophages appears as good mechanism aimed to reduce the symptoms observed during colitis.

In conclusion, here we show that Myo1F induces macrophage polarization to a proinflammatory phenotype by stimulating PI3K/Akt/STAT signaling. Myo1F deficiency reduces epithelial damage and stimulates recovery in a model of colitis DSS-induced.

## Author Contributions

ZP-Q designed research, performed research, analyzed data, and wrote the paper. CS performed research. SR-R performed ELISA assays. JM-M, MS, OM-C, and NV-S conducted experiments and interpreted data. PN and LS-A conceived and designed the research, interpreted data, and wrote the paper.

### Conflict of Interest Statement

The authors declare that the research was conducted in the absence of any commercial or financial relationships that could be construed as a potential conflict of interest.
