# Supplementary material for: Myosin 1F Regulates M1-Polarization by Stimulating Intercellular Adhesion in Macrophages
Source: Front Immunol. 2019 Jan 10;9:3118. doi: 10.3389/fimmu.2018.03118 (PMC6335276; doi:10.3389/fimmu.2018.03118)
Supplement: Supplementary file 1 [file Data_Sheet_1.pdf]

Supplementary figures 1

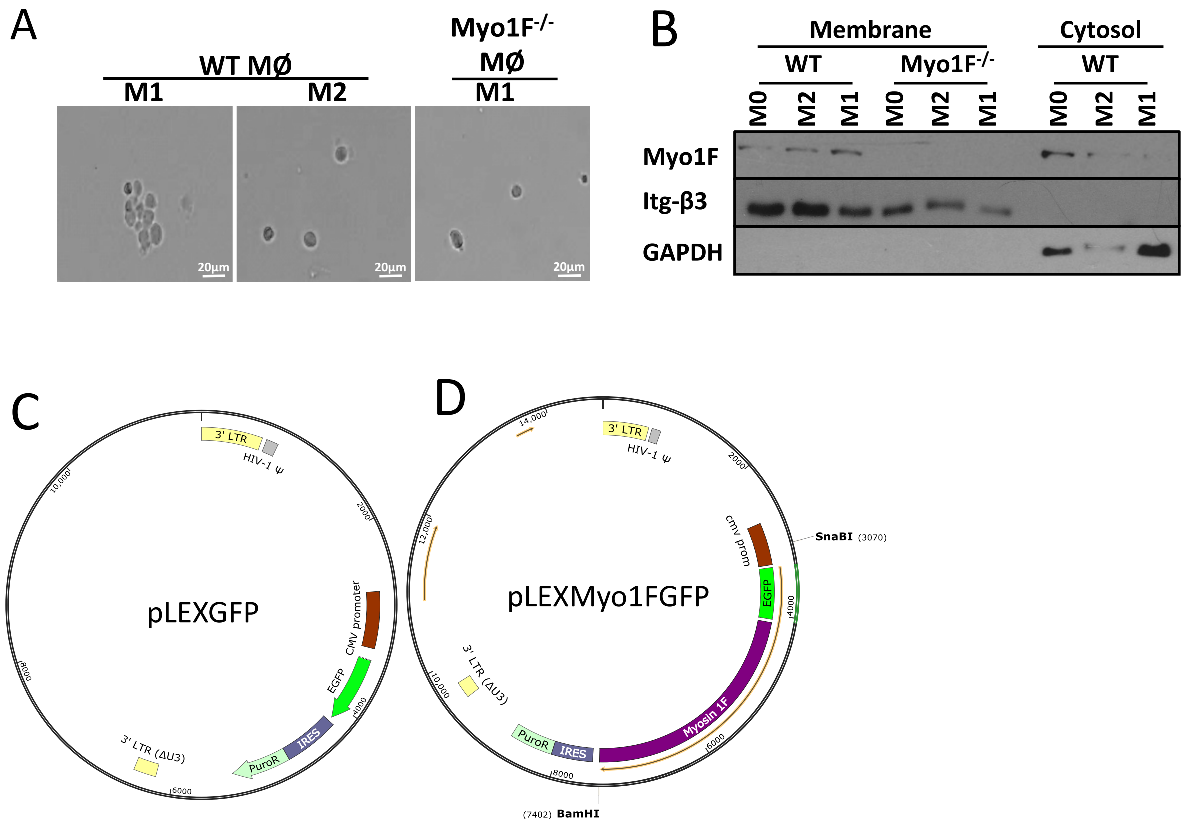

Figure S1. Lentiviral vector design.

A) Bright field images of BMM induced to M1 or M2 phenotype in a suspension assay. Bar= 20µm. n=5.

B) Membrane bound Myo1F was evaluated in BMM differentiated to M0, M1 and M2. Cytosolic and membrane fractions were prepared as previously reported (87). Integrin β3 marks membrane fraction and GAPDH = Cytosolic fraction.

C) Lentiviral vector pLEX expressing GFP reporter.

D) Lentiviral vector pLEX expressing myosin 1F protein fusioned to GFP in amine C-terminal end. Restriction enzymes SnaBI and BamHI were used to clone Myo1F to pLEX vector as describe in materials and methods.

Supplementary figure 2

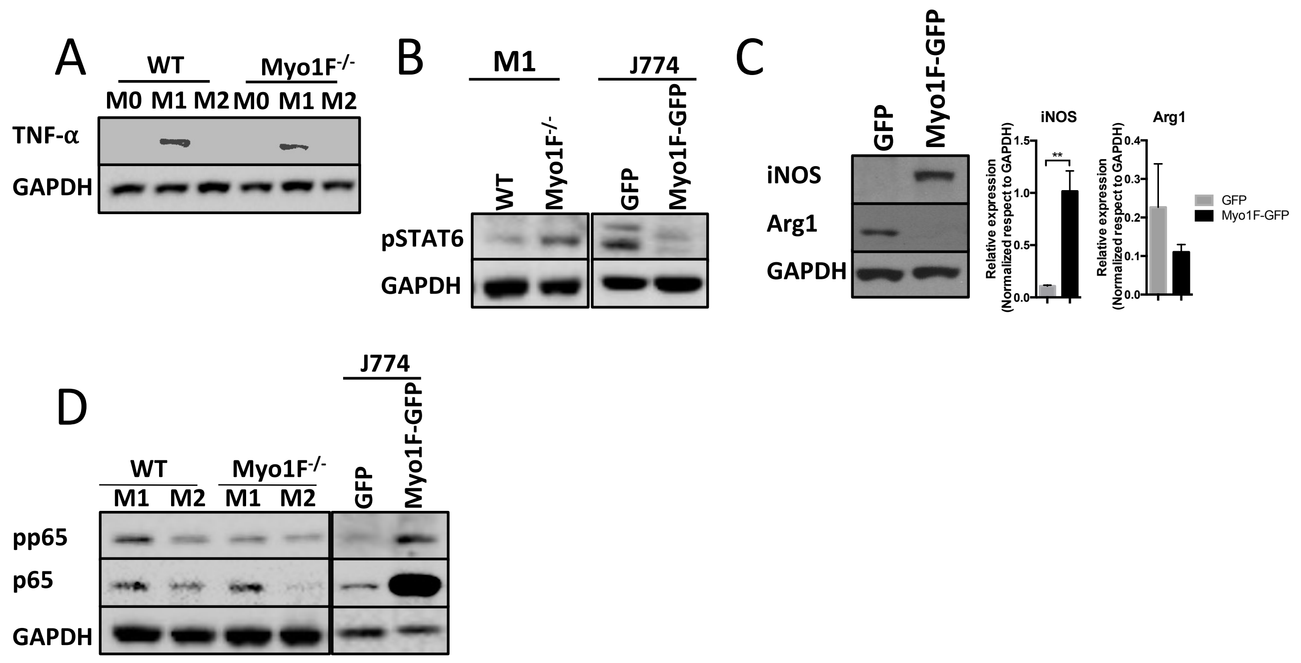

Figure S2. M1 phenotype is regulated by Myo1F.

A) Representative western blot for TNFα in cell lysates of M0, M1 and M2 BMM of WT and Myo1F$^{-/-}$ origin. GAPDH was used as loading control. n=3.

B) Representative western blot for pSTAT6 in cell lysates of M1 BMM of WT and Myo1F-/- origin and J774 transduced with GFP or Myo1F-GFP. GAPDH was used as loading control. n=3.

C) Representative western blot for iNOS and Arginase 1 (Arg1) in cell lysates of J774 transduced with GFP or Myo1F-GFP. Quantification is displayed in the graph. ***p*=0.01 GAPDH was used as loading control. n=6.

D) Representative western blot for pp65 and p65 in cell lysates of macrophages. BMM of WT and Myo1F-/- origin were differentiated into M1 and M2 phenotype. J774 stably transduced with GFP or Myo1F-GFP. GAPDH was used as loading control. n=3.

Supplementary figure 3

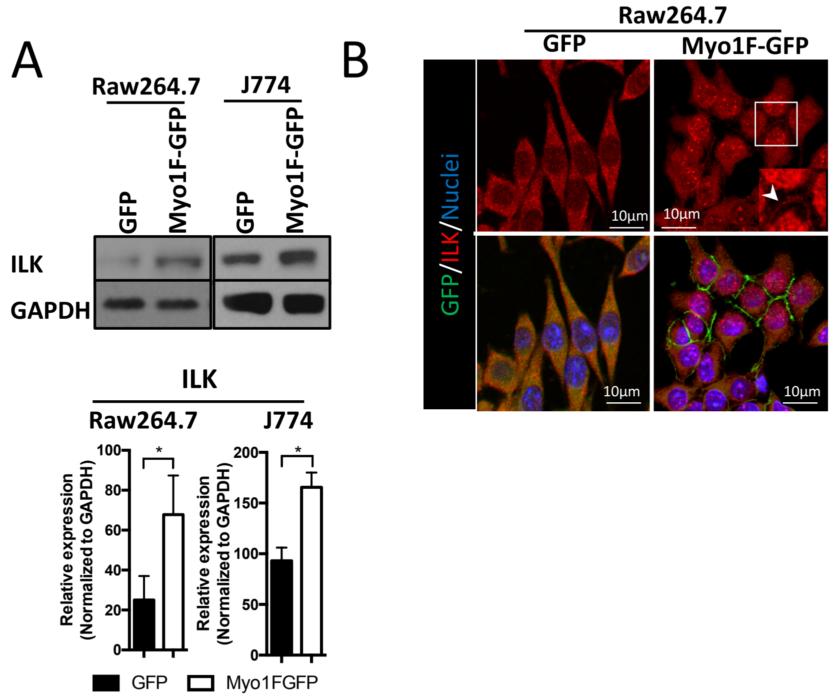

Figure S3. Myo1F positively regulates ILK and p65 signaling.

A) Representative western blot for ILK in cell lysates of RAW264.7 and J774 transduced with GFP or Myo1F-GFP. GAPDH was used as loading control. n=3. Graphs corresponding to densitometric analysis are shown. Mean derived from three independent experiments. **p*=0.05.

B) Immunofluorescence staining of ILK in RAW264.7 cells transduced with GFP or Myo1F-GFP virus. Inset depicts a cropped area marked with white square. White arrow = membrane bound ILK. GFP=Green. ILK=Red. Nuclei=Blue. n=5. Bar=10µm.

Supplementary figure 4

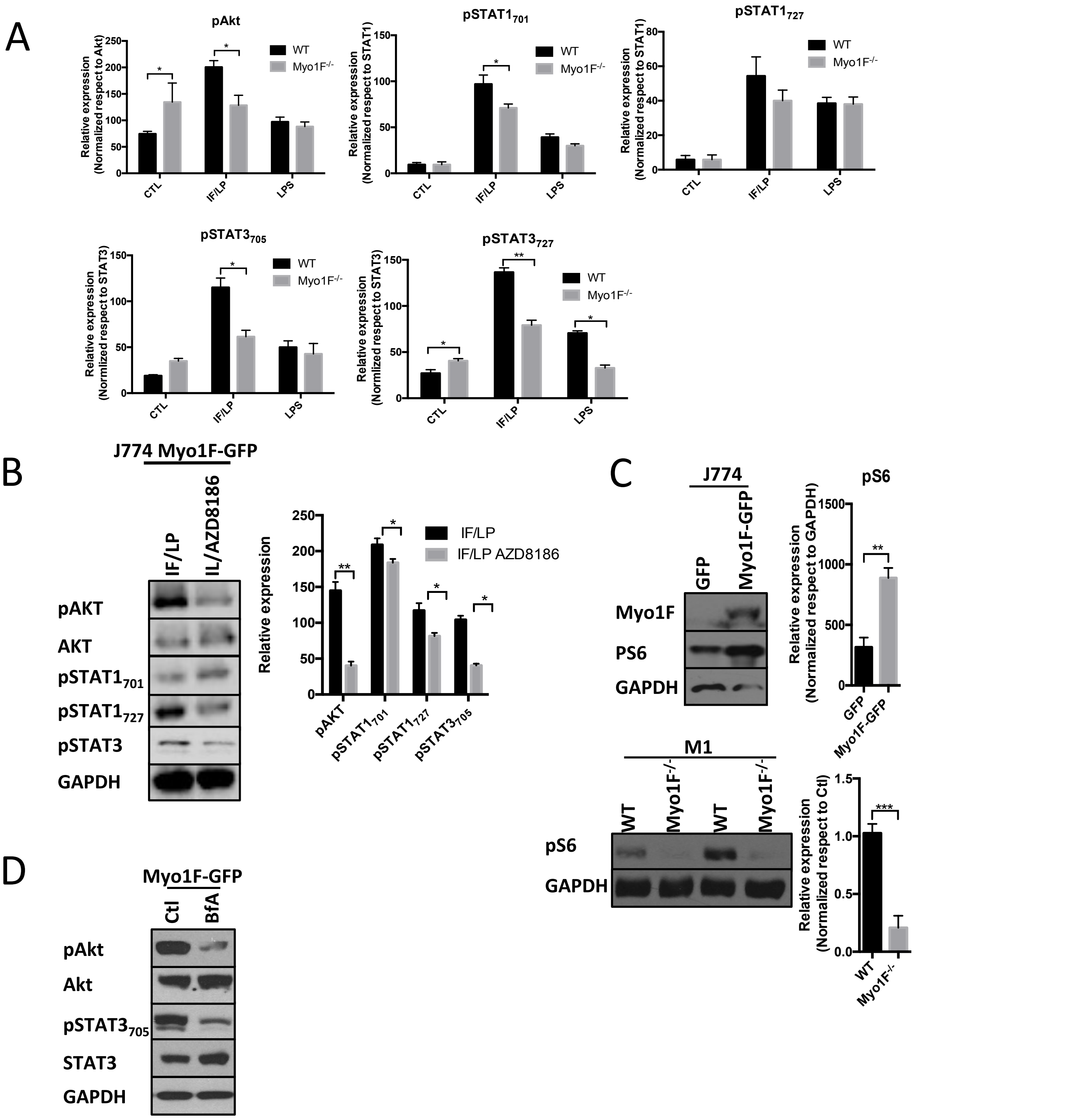

Figure S4. Inhibition of β3/PI3K/Akt/mTOR signaling prevents the increase in the activation of pSTAT1 and pSTAT3 but not STAT6 signaling.

A) Densitometric analysis were carried out after Western blotting pAkt473, pSTAT701, pSTAT727, pSTAT3705 and pSTAT3727 in cell lysates of WT or Myo1F$^{-/-}$ after LPS (1 μg/ml) or LPS (1μg/ml) plus IFN-γ (20ng/ml) stimulation for 5h. Phosphoproteins were normalized against total protein. Mean derived from three independent experiments is graph. **p*=0.05, ***p*=0.01.

B) Representative western blot for, pAkt, Akt, $pSTAT_{701}$, $pSTAT_{727}$, STAT1, pSTAT3 and STAT3 in cell lysates of J774 cells overexpressing Myo1F after IFN-γ (20ng/ml) priming for 1h plus 5h stimulation with LPS (1μg/ml) (IF/LP) or in presence of PI3K inhibitor (AZD8186). AZD8186 was administered 30 min before IF/LP stimulation. Densitometric analyses obtained from those results are shown as graphs. **p*=0.05, ***p*=0.01.

C) The mTORC1 marker, pS6, was analyzed in cell lysates of macrophages. Non-stimulated J774 overexpressing Myo1F or GFP were lysed in Ripa buffer. BMM of WT or Myo1F$^{-/-}$ were polarized to M1-phenotype with IFN/LPS for 24h. GAPDH was used as loading control. Densitometric analysis was carried out. n=3. ***p*=0.01, ****p*=0.0001.

D) pAkt473 and pSTAT3705 was analyzed by western blot in cell lysates of J774-Myo1F-GFP treated with Brefeldin A (BfA). BfA (10 μg/ml) treatment was carried out for 5h. n=3.

Supplementary figure 5

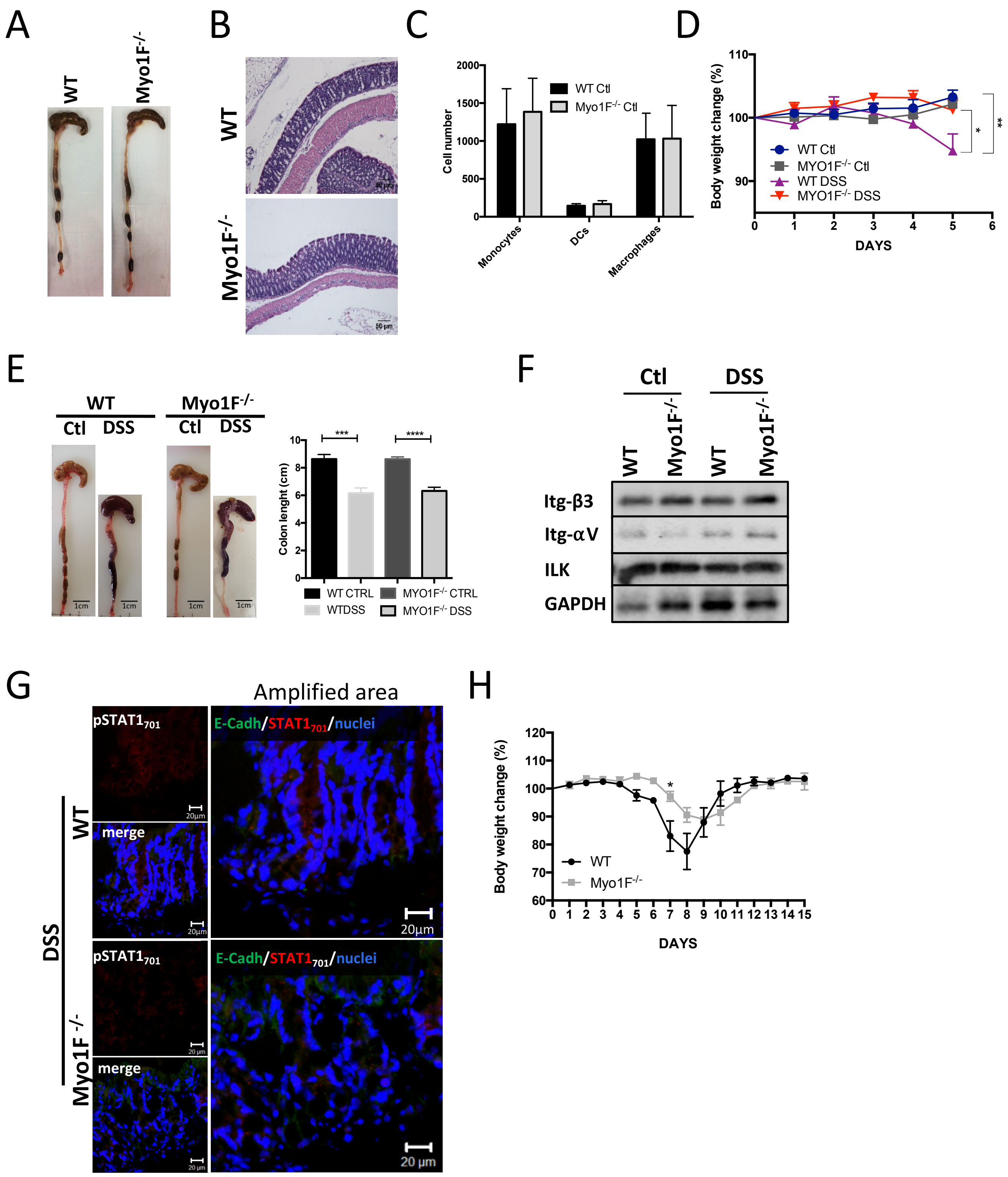

Figure S5. Myo1F regulates intestinal homeostasis.

A) Representative images depicting colon length of WT and Myo1F deficient mice in control conditions.

B) Hematoxylin & eosin staining of colonic sections obtained from WT and Myo1F deficient mice in control conditions.

C) Quantification of monocytes, macrophages and dendritic cells isolated from colon of WT and Myo1F$^{-/-}$ mice under control conditions. Purification and quantification was performed by Flow cytometry.

D) Percentage change in body weight in WT and Myo1F deficient mice induced to colitis.

E) Representative images depicting colon length and graphs showing quantification in WT and Myo1F deficient mice induced to colitis. DSS (2.5%) was dissolved in tap water and administered during 5 days. Control animals received water alone.
All the results are derived from independent experiments carry out by duplicate. n=3 mice per group. *p=0.05, **p=0.01, ***p=0.0005, ****p<0.0001. Scale bars: 1cm.

F) Western blot for integrin-αν, integrin-β3 and ILK was performed in cell lysates of colonic tissue obtained from WT and Myo1F deficient mice induced to colitis for 5 days. GAPDH was used as control loading.

G) Immunofluorescence staining for E-cadh (green) and pSTAT1, (red) in colonic epithelium from WT and Myo1F deficient mice after 5 days of treatment with DSS. Nuclei were stained with Dapi (blue). Magnification is shown. Bar=20 μm.

H) Percentage change in body weight in WT and Myo1F deficient mice in a recuperation assay after colitis induction. *p=0.05.
